# β1 integrins play a critical role maintaining vascular integrity in the hypoxic spinal cord, particularly in white matter

**DOI:** 10.1186/s40478-024-01749-4

**Published:** 2024-03-20

**Authors:** Sebok K. Halder, Arjun Sapkota, Richard Milner

**Affiliations:** San Diego Biomedical Research Institute, 3525 John Hopkins Court, Suite 200, 92121 San Diego, CA USA

**Keywords:** Spinal cord, Blood vessels, β1 integrin, Chronic mild hypoxia, White matter, Vascular integrity

## Abstract

Interactions between extracellular matrix (ECM) proteins and β1 integrins play an essential role maintaining vascular integrity in the brain, particularly under vascular remodeling conditions. As blood vessels in the spinal cord are reported to have distinct properties from those in the brain, here we examined the impact of β1 integrin inhibition on spinal cord vascular integrity, both under normoxic conditions, when blood vessels are stable, and during exposure to chronic mild hypoxia (CMH), when extensive vascular remodeling occurs. We found that a function-blocking β1 integrin antibody triggered a small degree of vascular disruption in the spinal cord under normoxic conditions, but under hypoxic conditions, it greatly enhanced (20-fold) vascular disruption, preferentially in spinal cord white matter (WM). This resulted in elevated microglial activation as well as marked loss of myelin integrity and reduced density of oligodendroglial cells. To understand why vascular breakdown is localized to WM, we compared expression levels of major BBB components of WM and grey matter (GM) blood vessels, but this revealed no obvious differences. Interestingly however, hypoxyprobe staining demonstrated that the most severe levels of spinal cord hypoxia induced by CMH occurred in the WM. Analysis of brain tissue revealed a similar preferential vulnerability of WM tracts to show vascular disruption under these conditions. Taken together, these findings demonstrate an essential role for β1 integrins in maintaining vascular integrity in the spinal cord, and unexpectedly, reveal a novel and fundamental difference between WM and GM blood vessels in their dependence on β1 integrin function during hypoxic exposure. Our data support the concept that the preferential WM vulnerability described may be less a result of intrinsic differences in vascular barrier properties between WM and GM, and more a consequence of differences in vascular density and architecture.

**Supplementary Information:**

The online version contains supplementary material available at 10.1186/s40478-024-01749-4.

## Introduction

In the central nervous system (CNS), blood vessels are particularly well adapted to protect vulnerable neural cells from potentially toxic blood components and to carefully regulate the selective transport of energy substrates (glucose, ketone bodies, amino acids) essential for neural function. Compared to blood vessels in other organs, CNS blood vessels display high electrical resistance and low permeability, and these characteristics are referred to as the blood-brain barrier (BBB), which physically separates the CNS from the blood [2, 20]. A variety of molecular mechanisms contribute to BBB integrity, including endothelial adhesive interactions with the underlying extracellular matrix (ECM) proteins of the vascular basement membrane (BM), tight junction protein complexes that connect adjacent endothelial cells, as well as the influence of CNS-resident cells such as astrocytes, pericytes, and microglia, which lie in close contact with vascular structures [4, 8, 13, 38]. While the CNS encompasses both the brain and spinal cord, recent studies have reported that the properties of blood vessels in these two different CNS compartments may be similar in many ways but also show some important differences. For instance, it has been reported that spinal cord blood vessels show increased permeability to a variety of substances including mannitol, inulin, interferons, sucrose, and tumor necrosis factor (TNF)-α [5, 28, 29, 31], implying that integrity of spinal cord vessels is less tight than the BBB, possibly due to reduced expression of tight junction proteins [12, 36]. In the spinal cord, the BBB is referred to as the blood-spinal cord barrier (BSCB) and its disruption has been described in several neurological diseases including multiple sclerosis (MS), amyotrophic lateral sclerosis (ALS), and spinal cord injury (SCI) [6, 11, 27, 35].

β1 integrins are a major class of cell surface receptors that mediate the influence of ECM proteins in regulating cell adhesion, proliferation, migration, and differentiation [21]. β1 integrins comprise more than 10 members, each with its own ligand specificity for individual ECM proteins. Within blood vessels, endothelial cells attach to a vascular BM that contains several ECM components, of which the main ones are laminin, collagen IV, fibronectin, and perlecan [8]. The ECM plays essential roles in regulating vascular growth and stabilization, both during development and in the adult [7, 9, 33]. More than 20 years ago, we made the observation that during development, cerebral blood vessels switch from expressing high levels of fibronectin and its cognate receptor α5β1 integrin during the angiogenic phase, to high levels of laminin and its cognate receptor α6β1 integrin at later timepoints, when the newly-formed blood vessels are undergoing maturation [25].

To understand how β1 integrins regulate angiogenic remodeling and vascular integrity in the brain, we recently examined the impact of a β1 integrin function-blocking antibody, both under normoxic stable conditions and during exposure to chronic mild hypoxia (CMH; 8% O_2_), when a strong vascular remodeling response occurs, resulting in a 50% increased cerebrovascular vessel density over two weeks [14, 23]. This showed that β1 integrin inhibition triggered low levels of BBB disruption under normoxic (control) conditions, but this disruptive effect was greatly amplified under hypoxic conditions, when cerebral blood vessels undergo extensive remodeling [18]. As growing evidence suggests that fundamental differences exist between brain and spinal cord blood vessels, both in terms of relative permeability, and expression of tight junction proteins [5, 28, 29, 31], this raises the possibility that blood vessels in these two CNS regions may be uniquely different in their dependence on specific barrier-related molecular mechanisms, including β1 integrins. Furthermore, as the spinal cord is cleanly demarcated into separate anatomical regions of white matter (WM) and grey matter (GM), this affords us the added advantage of being able to compare the vascular responses in these two compartments. Building on these findings, the aim of the current study was to evaluate the impact of β1 integrin blockade on vascular integrity in the WM and GM regions of the spinal cord, and determine how loss of this integrity affects microglial activation state and oligodendrocyte density.

## Materials and methods

### Animals

The studies described were reviewed and approved by the Institutional Animal Care and Use Committee at San Diego Biomedical Research Institute (SDBRI). Young female C57BL6/J mice were obtained from Jackson Laboratories and were maintained under pathogen-free conditions in the closed breeding colony of SDBRI.

### Chronic hypoxia model

Female C57BL6/J mice, 8–10 weeks were housed 4 to a cage, and placed into a hypoxic chamber (Biospherix, Redfield, NY) maintained at 8% O_2_ for 4 days. Littermate control mice were kept in the same room under similar conditions except that they were kept at ambient sea-level oxygen levels (normoxia, approximately 21% O_2_ at sea-level) for the duration of the experiment. Every few days, the chamber was briefly opened for cage cleaning and food and water replacement as needed.

### Administration of β1 integrin blocking antibody

Mice received daily intraperitoneal (i.p.) injections of either the anti-mouse β1 integrin function-blocking antibody (clone HMβ1-1) or an isotype control antibody (clone Ha4/8) both at doses of 2.5 mg/kg (BD Bioscience, La Jolla, CA).

### Immunohistochemistry and antibodies

Immunohistochemistry was performed on 10 μm frozen sections of cold phosphate buffer saline (PBS) perfused tissues as described previously [3]. Monoclonal antibodies from BD Biosciences reactive for the following antigens were used in this study: CD31 (clone MEC13.3; 1:300), Mac-1 (clone M1/70; 1:50), CD68 (clone FA-11; 1:2000), and the integrin subunits α5 (clone MFR5; 1:100), α6 (clone GoH3; 1:500) and β1 (clone 9EG7; 1:100). The hamster anti-CD31 (clone 2H8; 1:500) monoclonal was obtained from Abcam (Cambridge, MA). Rat anti-TER-119 (1:2000) and goat anti-PDGFRα (1:1000) were obtained from R&D systems. Rat anti-HSPG-2 (perlecan) (1:10000) was obtained from Chemicon. Rat anti-CD13 (1:1000) was obtained from Bio-Rad. Rat anti-PDGFRβ (1:400) was obtained from eBioscience. Rabbit antibodies reactive for the following proteins were also used: Ki67 (1:4000 from Novus Biologicals, Centennial, CO), fibronectin (1: 1000 from Sigma, St. Louis, MO), laminin (1: 1500 from Sigma), fibrinogen (1:1500 from Millipore, Temecula, CA), and claudin-5 (1:1500), occludin (1:2000), and ZO-1 (1:1500) all from Invitrogen, Carlsbad, CA. Goat antibodies used included anti VE-cadherin antibody (1:300 from R&D Systems) and anti-collagen IV (1:2000 from Sigma). Sheep anti-fibrinogen antibody (1:3000) was obtained from Bio-Rad. Mouse anti-β-dystroglycan (clone 43DAG/8D5; 1:500) was obtained from Novocastra, Newcastle-upon-Tyne, England. Mouse monoclonal anti-APC antibody (clone CC-1; 1:2000) was obtained from Sigma-Aldrich. Secondary antibodies used (all at 1:500) included Cy3-conjugated anti-rabbit, anti-rat, anti-hamster, and anti-sheep, Cy5-conjugated anti-rabbit from Jackson Immunoresearch, (West Grove, PA) and Alexa Fluor 488-conjugated anti-rat and anti-hamster from Invitrogen (Carlsbad, CA). Fluoromyelin red (1:50) was obtained from Invitrogen (Carlsbad, CA).

### Hypoxyprobe

The appearance of hypoxia in the spinal cord was detected by administration of hypoxyprobe (Hypoxyprobe Inc., Burlington, MA), consisting of pimonidazole hydrochloride which forms protein adducts in hypoxic cells when oxygen levels dip below 1.5% oxygen (pO_2_ < 10 mmHg). Hypoxyprobe was administered to mice by i.p. injection two hours before mice were removed from the hypoxia chamber and euthanized. Hypoxyprobe protein adducts were then detected using a rabbit polyclonal antibody according to the manufacturer’s instructions.

### Image analysis

Images were taken using a 5X, 10X or 20X objective on an Axioskop2 plus microscope (Carl Zeiss, Dublin, CA, USA) equipped with an Infinity 3 S camera (Lumenera, Ottawa, ON, Canada) and Infinity Analyze imaging software (Lumenera). For each antigen in all analyses, images of at least three randomly selected areas were taken at 5X, 10X or 20X magnification per tissue section and three sections per brain analyzed to calculate the mean for each animal (*n* = 4–10 mice per group). For each antigen in each experiment, exposure time was set to convey the maximum amount of information without saturating the image and was maintained constant for each antigen across the different experimental groups. The number of vascular leaks per field of view (FOV) in the spinal cord was quantified by capturing images and performing manual counts of the number of extravascular fibrinogen leaks. Total vascular leak area was measured and analyzed using NIH Image J software. In the brain (cerebellum and forebrain) we calculated the number of vascular leaks per unit area to adjust for the fact that the GM areas in the cerebellum or cerebral cortex are far greater than the WM areas in the cerebellum or corpus callosum. The number of activated microglia was quantified by performing manual counts of the number of CD68 ^+^ cells or by morphological criteria of Mac-1 staining (large cell body and short process extensions) per FOV. The number of mature oligodendrocytes and OPCs was quantified by counting the number of CC1 ^+^ or PDGFRα ^+^ cells/FOV respectively. Endothelial and microglial proliferation were quantified by counting the number of CD31/Ki67 or Mac-1/Ki67 dual-positive cells per FOV, respectively. Each experiment was performed with 4–10 different animals per condition, and the results expressed as the mean ± SEM. Statistical significance was assessed using one-way analysis of variance (ANOVA) followed by Tukey’s multiple comparison post-hoc test or Student’s t test, in which *p* < 0.05 was defined as statistically significant.

## Results

### β1 integrin inhibition greatly increases hypoxia-induced spinal cord vascular disruption preferentially in white matter

**Fig. 1:**
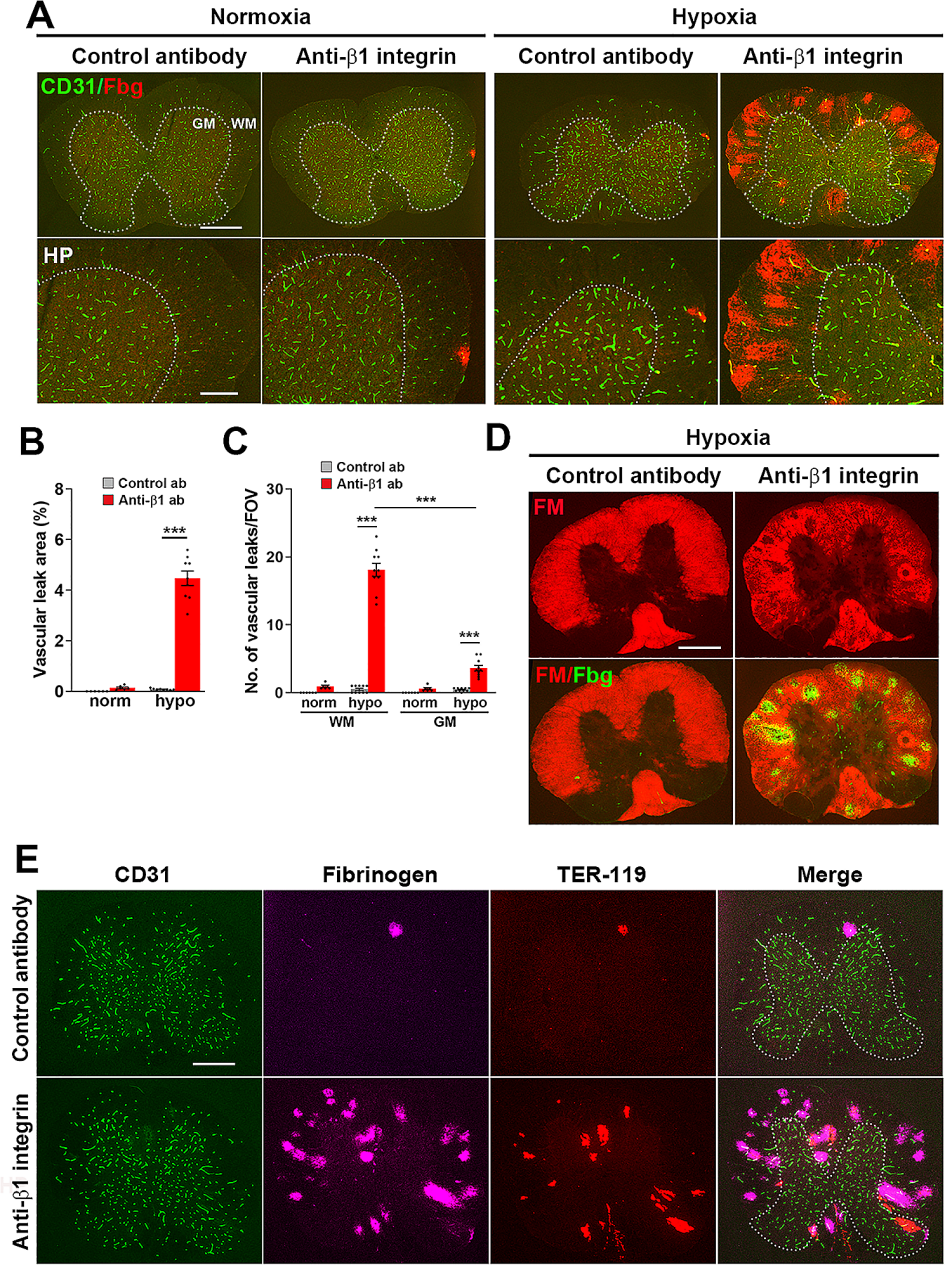
β1 integrin inhibition greatly increases hypoxia-induced spinal cord vascular disruption, preferentially in WM. Frozen spinal cord sections taken from mice exposed to normoxia or hypoxia (8% O_2_) that had received daily intraperitoneal injections of the anti-mouse β1 integrin function-blocking antibody or isotype control antibody for 4 days were stained for CD31 (AlexaFluor-488) and fibrinogen (Cy-3) (**A**), fluoromyelin (red) and fibrinogen (AlexaFluor-488) (**D**), or CD31 (AlexaFluor-488), fibrinogen (Cy-5), or TER-119 (Cy-3) (**E**). Scale bars = 500 μm, except for high power (HP) images in lower panel of A, where scale bar = 200 μm. White dotted line demarcates the GM (inside) from the WM (outside). (**B-C**) Quantification of the total area (**B**) or number of vascular leaks/FOV (**C**) in the spinal cord after normoxia or 4-days hypoxia. Results are expressed as the mean ± SEM (*n* = 6–10 mice/group). *** *p* < 0.001. Note that β1 integrin inhibition greatly increased the extent of hypoxia-induced vascular disruption in the spinal cord, preferentially in the WM

To evaluate how inhibition of β1 integrin function impacts vascular integrity of spinal cord blood vessels, young (8–10 weeks) mice were exposed to chronic mild hypoxia (CMH, 8% O_2_) or normoxic control conditions for 4 days, and received either daily intraperitoneal (i.p.) injections of the anti-mouse β1 integrin function-blocking antibody HMβ1-1, or an isotype control antibody (at doses of 2.5 mg/kg) for the duration of the 4 day CMH treatment. To confirm that the hamster anti-mouse β1 integrin blocking antibody localizes to blood vessels in spinal cord tissue, we conducted immunofluorescent (IF) studies with an anti-hamster secondary antibody. This demonstrated that the hamster blocking antibody localized very specifically to blood vessels (Supplementary Fig. 1). Vascular leak was evaluated by dual-IF using CD31 to identify endothelial cells and fibrinogen to detect extravascular leak. As shown in Fig. 1A, under normoxic conditions, while no spinal cord vascular leak was detected in mice receiving isotype control antibody, β1 integrin blockade triggered a small number of vascular leaks. However, under hypoxic conditions, while a relatively small number of leaks occurred in control antibody treated mice, β1 integrin inhibition greatly enhanced both the total number ($\sim$22-fold) and area ($\sim$65-fold) of spinal cord vascular leaks (Fig. 1A-C). Strikingly, most of these leaks occurred in the WM (shown in Fig. 1A and quantified in Fig. 1C). Dual-IF with fibrinogen and the WM marker fluoromyelin confirmed that almost all the hypoxia-induced vascular leaks occurred in the myelinated WM region of spinal cord (Fig. 1D). Of note, areas of vascular leak were strongly associated with marked loss of fluoromyelin signal, implying that vascular disruption erodes myelin integrity. Furthermore, triple-IF with CD31, fibrinogen, and the erythrocyte marker TER-119 demonstrated that many of these vascular disruptions were severe enough to result in hemmorhage into surrounding tissue, as shown by the extravascular leak of TER-119^+^ erythrocytes. In line with the fibrinogen data, most of these hemorrhages were also restricted to WM (Fig. 1E). From these findings we conclude first, that β1 integrins play an essential role in the maintenance of vascular integrity in spinal cord blood vessels, second, that this function is particularly critical during hypoxia-induced vascular remodeling, and third, this function appears to be especially relevant to WM blood vessels. To examine how β1 integrin inhibition impacts endothelial proliferation in spinal cord blood vessels, we performed dual-IF with CD31 and the proliferation marker Ki67 in the same group of mice. Consistent with our previous findings [17], this revealed that CMH strongly promoted endothelial proliferation in the spinal cord, with WM showing the strongest response (Supplementary Fig. 2B). Of note, β1 integrin blockade had no obvious effect on the rate of endothelial proliferation in the hypoxic spinal cord, either in WM or GM (Supplementary Fig. 2).

### β1 integrin inhibition greatly enhances hypoxia-induced microglial activation

**Fig. 2:**
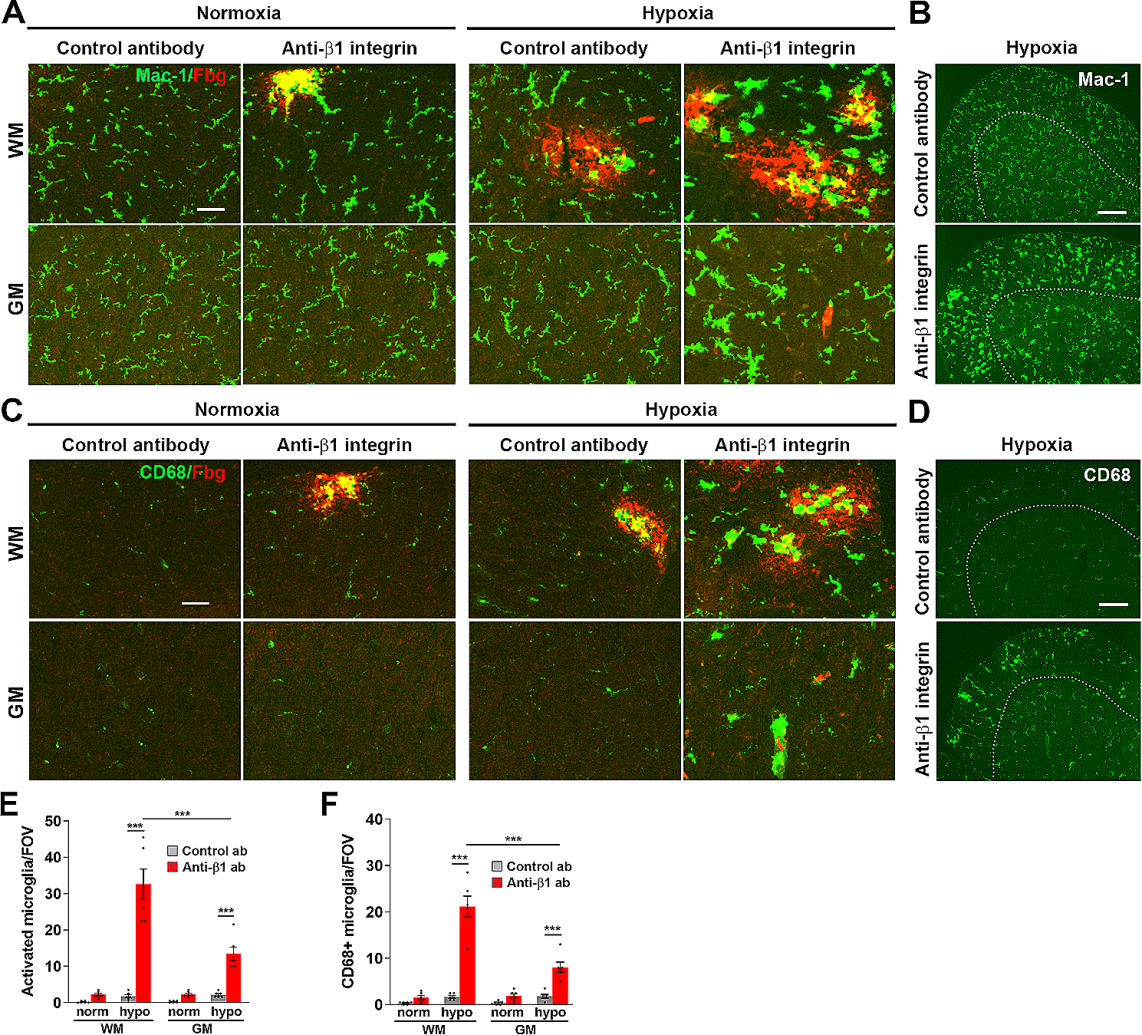
β1 integrin inhibition greatly enhances microglial activation in the hypoxic spinal cord. Frozen spinal cord sections taken from mice exposed to normoxia or hypoxia (8% O_2_) that had received daily intraperitoneal injections of the anti-mouse β1 integrin function-blocking antibody or isotype control antibody for 4 days were stained for Mac-1 (AlexaFluor-488) and fibrinogen (Cy-3) (**A**) or CD68 (AlexaFluor-488) and fibrinogen (Cy-3) (**C**). Lower magnification images of Mac-1 and CD68 IF are shown in B and D, respectively. Scale bars = 50 μm (A and C) or 200 μm (B and D). White dotted line demarcates the GM (inside) from the WM (outside). Quantification of the number of morphologically activated microglia/FOV (**E**) or number of CD68 ^+^ microglia/FOV (**F**) after 0- or 4-days hypoxia. Results are expressed as the mean ± SEM (*n* = 6 mice/group). *** *p* < 0.001. Note that β1 integrin inhibition strongly increased all parameters of microglial activation in the hypoxic spinal cord

As we have shown that hypoxia-induced CNS vascular disruption results in a marked microglial activation response [13–15], we next investigated how β1 integrin inhibition impacts microglial activation in the spinal cord by performing dual-IF with Mac-1/fibrinogen and CD68/fibrinogen. This showed that under normoxic conditions, Mac-1^+^ microglia occupied a ramified inactivated morphology (Fig. 2A), along with low levels of CD68 (a marker of microglial priming; Fig. 2C). Under normoxic conditions, β1 integrin inhibition induced a very small, but non-significant increase in these markers. Under hypoxic conditions, the isotype control antibody also triggered a small non-significant upwards trend of microglial activation markers compared to the normoxic baseline, but most strikingly, β1 integrin inhibition triggered a very strong microglial activation response in both the WM and GM compartments of spinal cord as shown by marked morphological transformation into the classical activated phenotype (large cell body with short processes) and increased number and size of CD68^+^ cells (Fig. 2A-B). As microglial proliferation is an integral part of a strong microgliosis response, we also evaluated this by Mac-1/Ki67 dual-IF (supplementary Fig. 3). This showed that the density of proliferating microglia was very low under normoxic conditions. After hypoxic exposure, while proliferating microglia were rarely present under isotype control antibody conditions, this number was strongly increased by β1 integrin inhibition, indicating a strong microglial proliferation response (see arrows in Supplementary Fig. 3A). Notably, enhancement of microglial activation was strongest in the WM (Fig. 2E-F and Supplementary Fig. 3B), concordant with the greater levels of vascular disruption in this region.

### β1 integrin inhibition triggers loss of oligodendroglial cells specifically in WM

**Fig. 3:**
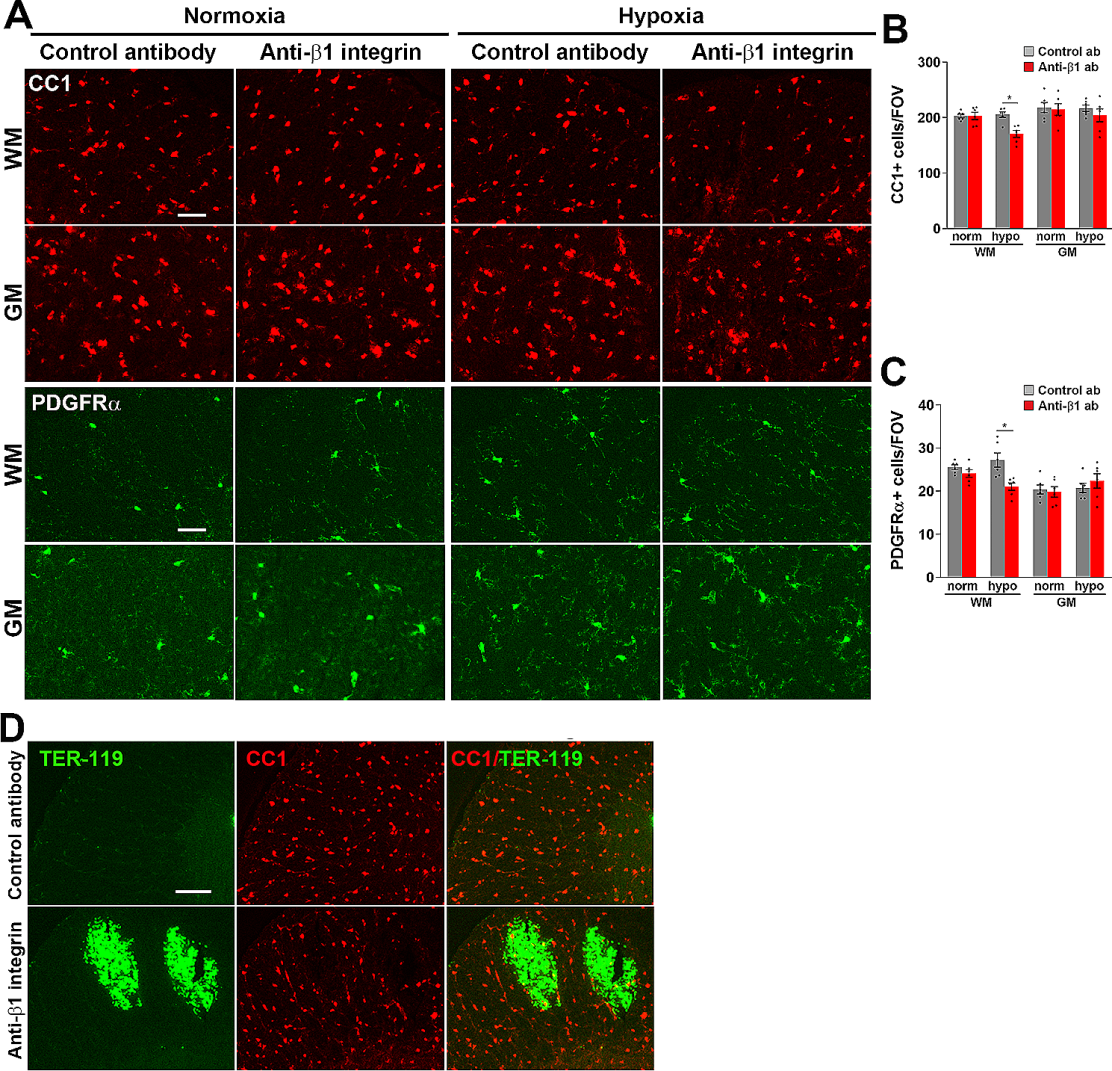
β1 integrin inhibition triggers loss of oligodendroglial cells specifically in WM. Frozen spinal cord sections taken from mice exposed to normoxia or hypoxia (8% O_2_) that had received daily intraperitoneal injections of the anti-mouse β1 integrin function-blocking antibody or isotype control antibody for 4 days were stained for CC1 (top two rows)) or PDGFRα (lower two rows) (**A**) or TER-119 (AlexaFluor-488) and CC1 (Cy-3) (**D**). Scale bars = 50 μm (**A**) or 100 μm (D). Quantification of the number of CC1 ^+^ cells/FOV (**B**) or PDGFRα ^+^ cells/FOV (**C**) after 0- or 4-days hypoxia. Results are expressed as the mean ± SEM (*n* = 6 mice/group). * *p* < 0.05. Note that β1 integrin inhibition decreased the cell density of mature oligodendrocytes and OPCs in the hypoxic spinal cord, specifically in the WM. Also note that vascular disruption led to obvious loss of oligodendrocytes in the immediate vicinity of erythrocyte deposition (**D**)

Because fluoromyelin/fibrinogen dual-IF indicated a strong correlation between vascular leak and myelin degradation (Fig. 1D), we next evaluated the impact of β1 integrin inhibition on cells of the oligodendroglial lineage. First, we quantified by IF the cell density of mature oligodendrocytes using the CC1 marker (Fig. 3A). This revealed that hypoxia alone had no noticeable impact on the density of CC1 ^+^ cells, and that under normoxic conditions, β1 integrin inhibition had no discernible influence on CC1 ^+^ cell density. However, in the presence of hypoxia, β1 integrin inhibition significantly reduced the density of CC1^ +^ cells specifically in the WM (Fig. 3A-B). We next examined how β1 integrin inhibition influences the density of oligodendrocyte precursor cells (OPCs) by staining for platelet derived growth factor receptor alpha (PDGFRα). In a similar manner to CC1, PDGFRα IF revealed that hypoxia alone did not affect the density of OPCs, and that under normoxic conditions, β1 integrin inhibition had no discernible influence on OPC density, but in the presence of hypoxia, β1 integrin inhibition significantly reduced the density of OPCs specifically in the WM (Fig. 3C). These data are consistent with our finding that under hypoxic conditions, β1 integrin inhibition triggers greatest vascular disruption in WM (Fig. 1), which in turn leads to greater loss of oligodendroglial cells in this region. Dual-IF with the erythrocyte marker TER-119 and the oligodendrocyte marker CC1 showed that in WM regions showing large vascular leaks, extravascular deposition of erythrocytes correlated with striking loss of oligodendrocytes (note the two dark holes corresponding to the TER-119^+^ region in the lower middle panel of Fig. 3D).

### In conditions of chronic mild hypoxia, spinal cord tissue hypoxia is most severe in the WM

Our observation that under hypoxic conditions, β1 integrin inhibition disrupts vascular integrity preferentially in WM demonstrates a novel and fundamental difference between WM and GM blood vessels. This raises the question: why are WM blood vessels so vulnerable to disruption while GM vessels are relatively resistant? One possibility is that the expression of molecules contributing to vascular integrity differs between WM and GM. For instance, if WM blood vessels express lower levels of β1 integrins, one might expect the blocking antibody would more effectively block β1 integrin function in WM before it does in GM. To address this possibility, we compared WM and GM for vascular expression of cell adhesion receptors (the integrin subunits β1, α5 and α6, and dystroglycan, Supplementary Fig. 4A), the ECM proteins fibronectin, laminin, collagen IV and perlecan (Supplementary Fig. 4B), tight junction proteins claudin-5, occludin, and ZO-1 and the junctional adhesion protein VE-cadherin (Supplementary Fig. 4C), and markers of pericyte coverage (CD13 and PDGFRβ, Supplementary Fig. 4D). This confirmed first that vascular density in GM is $\sim$22-fold) and area ($\sim$4 times higher than in WM, and second, that vascular expression of almost all molecules examined was noticeably higher in hypoxia treated mice both in the WM and GM regions. However, at the single vessel level, we did not observe any major WM/GM differences in the expression levels of any of these important BBB molecules.

**Fig. 4:**
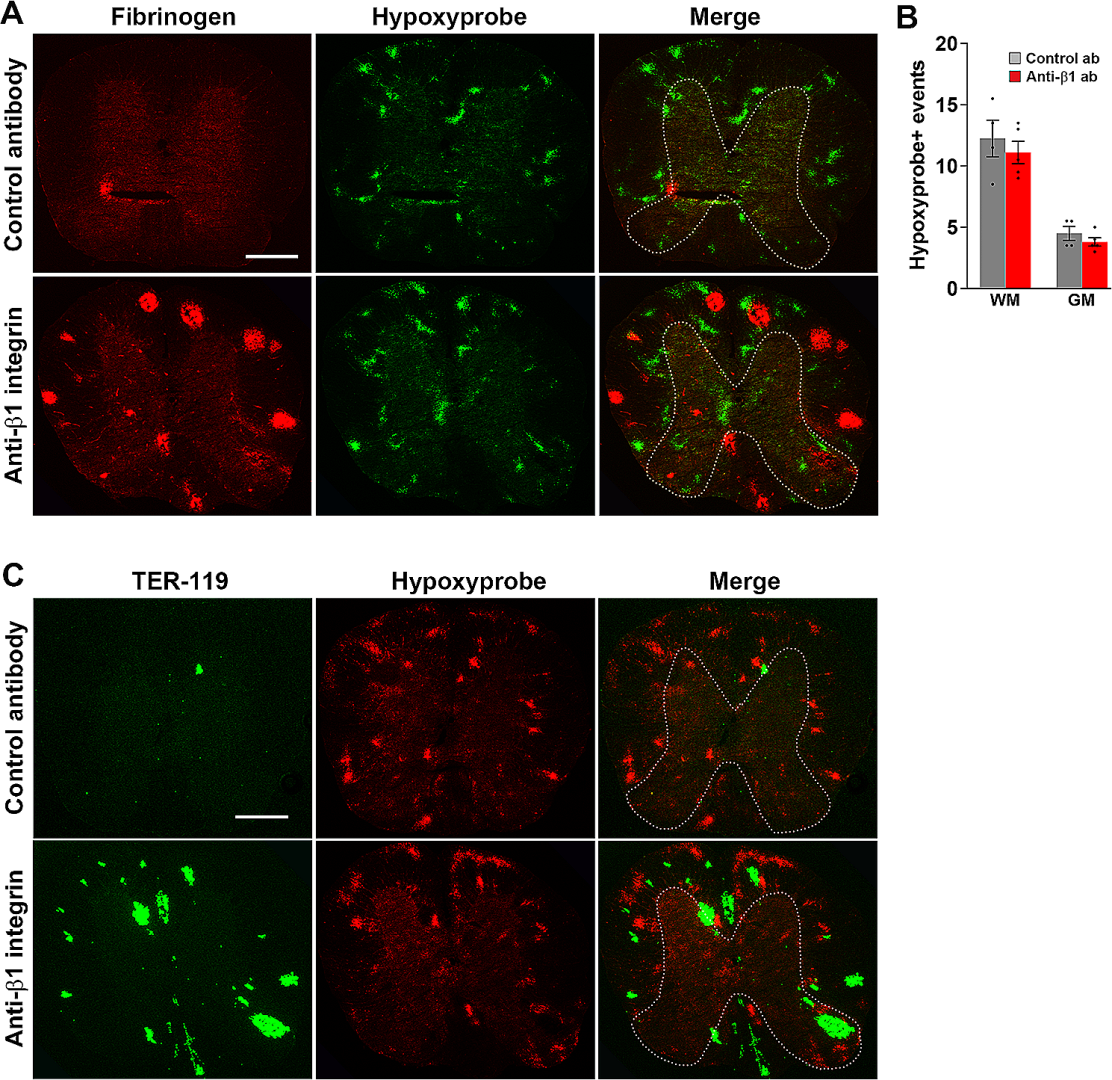
In the chronic mild hypoxia model, tissue hypoxia is most severe in the WM. Frozen spinal cord sections taken from mice exposed to hypoxia (8% O_2_) that had received daily intraperitoneal injections of the anti-mouse β1 integrin function-blocking antibody or isotype control antibody for 4 days, followed by an intraperitoneal injection of hypoxyprobe 2 h before tissue samples were collected, were stained for fibrinogen (Cy-3) and hypoxyprobe (AlexaFluor-488) (**A**), or TER-119 (AlexaFluor-488) and hypoxyprobe (Cy3) (**C**). Scale bars = 500 μm. White dotted line demarcates the GM (inside) from the WM (outside). **B**. Quantification of the number of hypoxyprobe ^+^ events/FOV in the spinal cord after 4-days hypoxia. Results are expressed as the mean ± SEM (*n* = 4–5 mice/group). Note that most hypoxyprobe ^+^ events occurred in the WM, that many vascular leaks, but not all, were associated with a hypoxyprobe ^+^ region, and that β1 integrin inhibition while greatly increasing the number of vascular leaks, had no effect on the appearance or distribution of hypoxic regions

An alternative possibility that might account for the greater vulnerability of WM vessels to hypoxia is that differences in vascular density or architecture are responsible. Specifically, vessel density in GM is 4 times greater than WM; thus, the average vessel to cell diffusion distance is 4 times greater in WM [17, 19, 34]. In keeping with this greater vascular density, cerebral blood flow has been shown to be 4 times greater in GM compared to WM [10, 24]. Based on these observations, it has been suggested that GM tissue has a much greater vascular reserve than WM [10, 24]. Thus, when oxygen is limited, WM tissue is far more likely to experience greater levels of hypoxia, and as hypoxia triggers angiogenesis and vascular breakdown, the result is more vascular disruption in WM. To directly test this idea in the CMH model, we evaluated the appearance of hypoxia in the spinal cord by i.p. administration of hypoxyprobe (pimonidazole hydrochloride), which forms permanent protein adducts in hypoxic cells when oxygen levels dip below 1.5% oxygen (pO_2_ < 10 mmHg), and which can subsequently be detected by polyclonal antibody [1, 32]. As expected, the normoxic spinal cord contained no hypoxyprobe^+^ regions or vascular leaks. In contrast, spinal cord sections taken from CMH mice contained many hypoxyprobe^+^ regions and importantly, the vast majority of these were in the WM (Fig. 4A-B). Interestingly, while β1 integrin inhibition did not change the density or WM/GM distribution of hypoxyprobe^+^ regions, it triggered a strong increase in the number of vascular leaks (Fig. 4A) and most of these were in the WM (Fig. 4B). Dual-IF with the erythrocyte marker TER-119 and hypoxyprobe confirmed that the majority of hypoxyprobe^+^ regions occur in the WM, and that hypoxia, superimposed with β1 integrin blockade, results in greatly increased numbers of vascular leaks, specifically in spinal cord WM (Fig. 4C-D). Together, these data show that in mice exposed to CMH, spinal cord WM is far more susceptible to manifesting regions of hypoxia and vascular disruption. Taken with our previous observation that WM launches a more vigorous angiogenic response to hypoxia than GM [17], we propose a model that explains all these findings: (i) the low vessel density (and blood flow) in WM predisposes to greater susceptibility to hypoxia, (ii) this hypoxia drives angiogenesis at a faster rate in WM, and (iii) the greater extent of angiogenic remodelling in WM, which involves uncoupling of endothelial cells from ECM proteins, allows the β1 blocking antibody greater access to interfere with endothelial-ECM interactions, resulting in higher levels of vascular disruption in WM.

### White matter tracts in the brain also show greater susceptibility to β1 integrin inhibition-induced vascular leak under hypoxic conditions

**Fig. 5:**
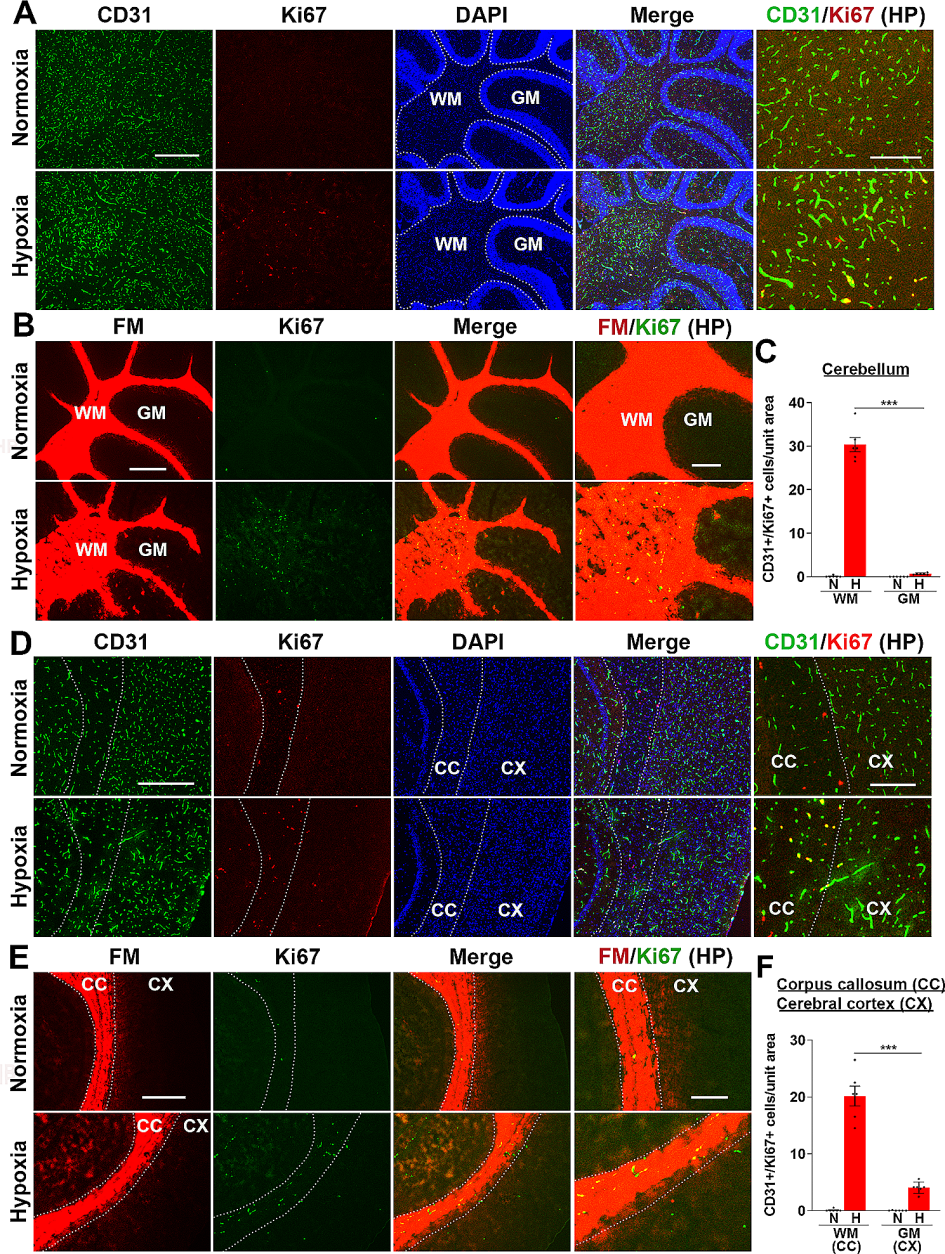
Hypoxia-induced endothelial proliferation in the brain occurs preferentially in white matter tracts. Frozen brain sections taken from mice exposed to normoxia or hypoxia (8% O_2_) for 4 days were stained for CD31 (AlexaFluor-488), Ki67 (Cy-3), and DAPI (blue) (**A**, cerebellum and **D**, forebrain) or fluoromyelin (red) and Ki67 (AlexaFluor-488) (**B**, cerebellum and **E**, forebrain). Scale bars = 500 μm except for high power (HP) images where scale bars = 200 μm. White dotted line demarcates the WM corpus callosum (CC, inside) from the surrounding cerebral cortex (CX) grey matter. **C** and **F**. Quantification of the number of proliferating endothelial cells/unit area in the cerebellum (**C**) or cerebral cortex/corpus callosum areas (**F**) after 0- or 4-days hypoxia. Results are expressed as the mean ± SEM (*n* = 5–6 mice/group). *** *p* < 0.001. Note that in both brain areas, most of the hypoxia-induced endothelial proliferation occurs in the WM tracts

**Fig. 6:**
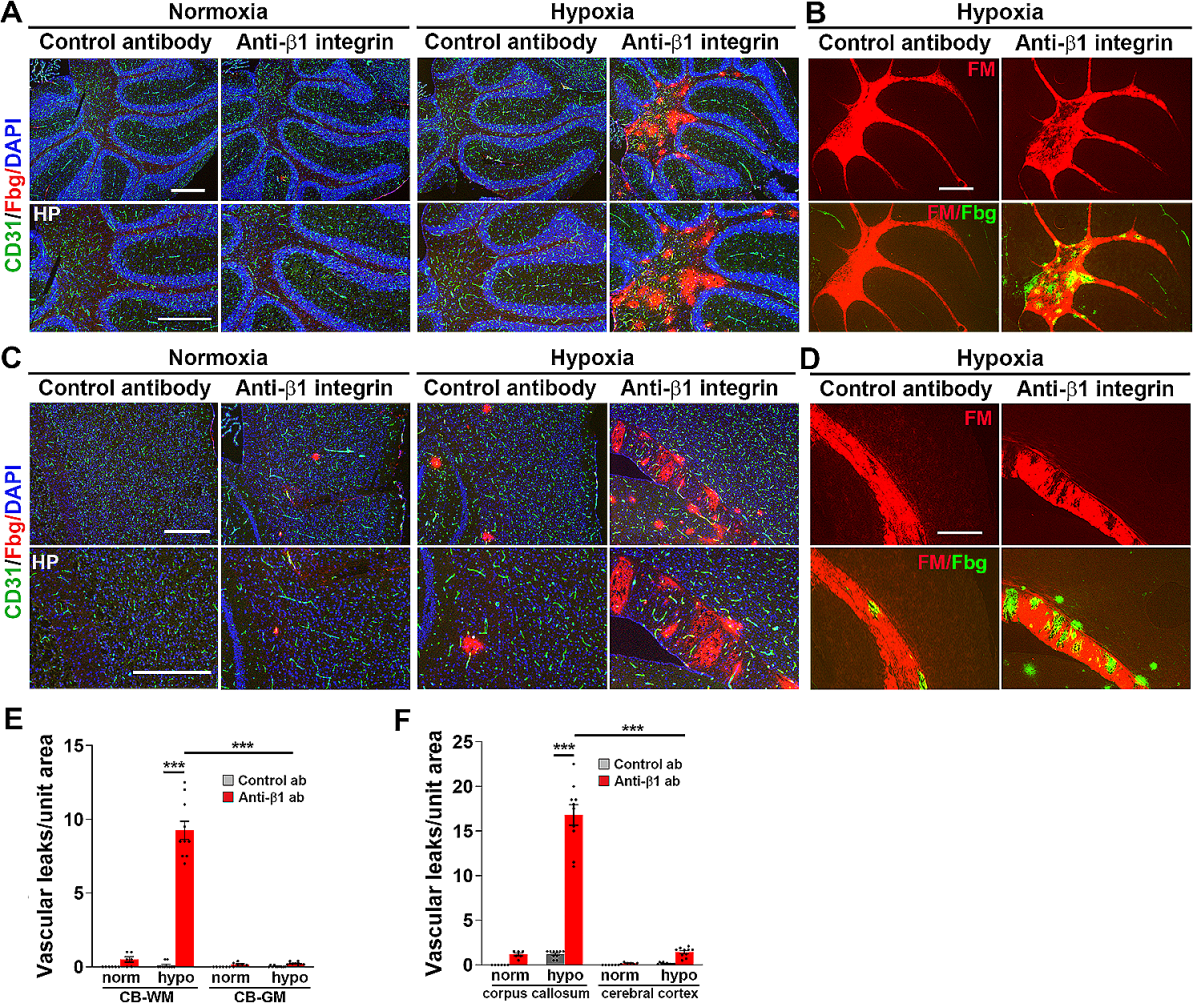
White matter tracts in the brain show a similar predilection to β1 integrin inhibition-induced vascular leak under hypoxic conditions. Frozen brain sections taken from mice exposed to normoxia or hypoxia (8% O_2_) that had received daily intraperitoneal injections of the anti-mouse β1 integrin function-blocking antibody or isotype control antibody for 4 days were stained for CD31 (AlexaFluor-488), fibrinogen (Cy-3) and DAPI (blue) (**A and C**) or fluoromyelin (red) and fibrinogen (AlexaFluor-488) **(B and D)**. A-B and C-D images show the cerebellum and cerebral cortex/corpus callosum, respectively. Scale bars = 500 μm. White dotted line demarcates the WM corpus callosum (inside) from the surrounding grey matter. **E-F**. Quantification of the number of vascular leaks/unit area in the cerebellum (E) or cerebral cortex/corpus callosum areas after 0- or 4-days hypoxia. Results are expressed as the mean ± SEM (*n* = 6–10 mice/group). *** *p* < 0.001. Note that under hypoxic conditions, β1 integrin inhibition greatly increased the extent of vascular disruption in the brain, preferentially in the cerebellar and corpus callosum WM tracts

Based on our spinal cord findings that β1 integrin inhibition greatly increases hypoxia-induced vascular disruption almost exclusively in WM, we next extended our analysis to the brain to see if our proposed model also holds true there. Here we examined two well demarcated WM tracts; the WM tracts of the cerebellum and the corpus callosum, and compared them with the surrounding GM areas of the cerebellum and cerebral cortex, respectively. First, we compared the angiogenic response in WM and GM regions by quantifying the density of proliferating endothelial cells, using CD31/Ki67 dual-IF. This showed that in the cerebellum, almost all the proliferating endothelial cells were in the WM tract, with very few in the neighboring GM (Fig. 5A). This was confirmed using fluoromyelin/Ki67 dual-IF (Fig. 5B). Quantification of CD31^+^/Ki67^+^ cells in the cerebellum revealed a WM:GM ratio of 43:1 (Fig. 5C). In a similar manner, in the forebrain, the WM corpus callosum tract contained a much higher density of proliferating endothelial cells than the neighboring cerebral cortex (Fig. 5D), which was confirmed by fluoromyelin/Ki67 dual-IF (Fig. 5E). Quantification of CD31^+^/Ki67^+^ cells in the WM corpus callosum and the GM cerebral cortex revealed a WM:GM ratio of 5:1, (Fig. 5F). Thus, consistent with our observations in spinal cord WM [17], in both these brain regions, the WM contained a much greater number of proliferating endothelial cells. Next, we compared the relative distribution of BBB disruption in the WM and GM regions of the cerebellum and the forebrain (cerebral cortex and corpus callosum). Similar to spinal cord, this revealed that under normoxic conditions, no BBB disruption was detected in mice receiving isotype control antibody, while β1 integrin blockade triggered a very small number of vascular leaks (Fig. 6). However, under hypoxic conditions, while a small number of leaks occurred in control antibody treated mice, β1 integrin inhibition greatly enhanced the number of vascular leaks both in the cerebellum and the forebrain ($\sim$75-fold and 13-fold respectively compared to isotype control conditions). Most strikingly, these vascular leaks occurred almost exclusively in the WM, both in the cerebellum (Fig. 6A) and the corpus callosum of the forebrain (Fig. 6C). In the cerebellum, almost all vascular leaks occurred in the WM tract, with very few in the surrounding GM (WM:GM ratio of 41:1, Fig. 6E), while the corpus callosum WM tract contained many vascular leaks with just a few in the surrounding cerebral cortex GM (WM:GM ratio of 12, Fig. 6F). Fluoromyelin/fibrinogen dual-IF confirmed that almost all vascular leaks identified in these brain regions occurred in the myelinated WM regions (Fig. 6B and D). In addition, in both the cerebellum and forebrain, fluoromyelin/fibrinogen dual-IF demonstrated a strong spatial association between vascular disruption and myelin degradation like that found in the spinal cord (note the motheaten appearance of myelin in Fig. 6B and D). These observations support the concept that the preferential tendency for WM (in both brain and spinal cord) to show vascular disruption in response to CMH in the presence of β1 integrin blockade, is a result of lower vascularity triggering stronger angiogenic remodelling, allowing the β1 integrin blocking antibody more opportunity to prevent the stabilization of newly formed blood vessels in these regions.

## Discussion

Motivated by our recent finding that β1 integrins play a critical role maintaining vascular integrity in the brain, particularly under hypoxia-induced vascular remodeling conditions [18], here we examined how β1 integrin inhibition impacts the vascular integrity of spinal cord blood vessels. This is important because (a) evidence suggests that brain and spinal cord vessels have fundamentally different properties [5, 28, 29, 31], and (b) the fact that spinal cord is cleanly demarcated into separate WM and GM regions affords us the opportunity to compare vascular properties in these two distinct regions. Our main findings were as follows. First, while β1 integrin inhibition triggered a small amount of spinal cord vascular disruption under normoxic conditions, under hypoxic conditions, when an active remodelling process is underway, it greatly enhanced (20-fold) spinal cord vascular disruption, and strikingly, most of this disruption occurred preferentially in the WM. Second, vascular disruption resulted in greatly enhanced microglial activation as well as marked loss of myelin integrity and reduced density of oligodendroglial cells. Third, comparison of vascular expression levels of the major BBB molecular components revealed no obvious differences between WM and GM blood vessels. Fourth, at the tissue level, the most severe levels of hypoxia induced by CMH occurred in spinal cord WM. Fifth, analysis of brain tissue, specifically the cerebellum and forebrain revealed a similar preferential vulnerability of WM tracts to show vascular disruption under these conditions. Taken together, these findings demonstrate an essential role for β1 integrins in maintaining vascular integrity in the spinal cord, and interestingly, reveal a novel and unexpected difference between WM and GM blood vessels in their dependence on β1 integrin function during hypoxic exposure. Specifically, the major implication is that compared to those in spinal cord GM, blood vessels in WM are much more dependent on β1 integrin function to promote vascular remodeling and stabilization during exposure to hypoxia and perhaps other modeling conditions such as neuroinflammation. They also suggest that this difference may be less a result of intrinsic differences in barrier properties but more a consequence of reduced vascular density in WM promoting a far stronger angiogenic response to hypoxic challenge, leading to vascular breakdown, microglial activation, and demyelination.

### β1 integrins are essential for spinal cord vascular integrity

While several studies have demonstrated the importance of β1 integrins in maintaining vascular integrity in blood vessels in the brain [22, 26, 39], to our knowledge, no-one has yet examined their role in spinal cord blood vessels. This becomes important in light of previous findings showing that vascular integrity of spinal cord vessels is lower than BBB integrity [5, 28, 29, 31], which may be attributable to reduced levels of pericyte coverage and endothelial expression of tight junction proteins [36]. Here we found that like our recently reported observations in the brain, inhibition of β1 integrins had a minor disruptive effect on vascular integrity during normoxic (control) conditions, but this was greatly magnified during hypoxic exposure, when a robust vascular remodeling response is underway. From a clinical perspective, as spinal cord vascular integrity declines during the pathological progression of amyotrophic lateral sclerosis (ALS) [37], it will be worth examining if downregulation of β1 integrins or their activation, is a feature of this reduced vascular integrity.

### Why are WM blood vessels particularly sensitive to β1 integrin inhibition?

The findings presented here show that just as in the brain [18], β1 integrins play an important role in stabilizing vessel stability in the spinal cord, and that while this is less evident under normoxic stable conditions, it becomes very apparent during hypoxia-induced vascular remodeling. The most likely reason for this is that in contrast to normoxic conditions, when endothelial cells and their integrin-ECM interactions are relatively stable and inaccessible to the β1 integrin antibody, during vascular remodeling, endothelial cells are in a state of flux and integrin-ECM interactions are making and breaking, which permits the β1 integrin antibody much greater opportunity to interfere with these cell-ECM interactions. By the same reasoning, we propose that this also underlies the greater susceptibility of WM vessels to leak. As blood vessel density in WM is only one fourth that of GM, it means that in times of reduced oxygen availability, WM tissue is far more likely to experience greater levels of hypoxia, which will trigger a stronger angiogenic response in this region. This concept is supported by (i) our current finding that most of the hypoxyprobe^+^ regions are found in the WM and (ii) our observation that in both spinal cord and brain, WM show higher rates of hypoxia-induced angiogenesis compared to GM [17]. These events will allow the β1 integrin antibody more opportunity to interfere with vascular migration and maturation, culminating in greater levels of vascular disruption in WM. In addition to the influence of vascular density in accounting for WM’s heightened susceptibility to hypoxic disruption, it is also possible that differences in vascular architecture may play a role. Specifically, while GM contains abundant numbers of arterioles, these are very rare in WM, raising the possibility that in contrast to GM capillaries, which are close to their feeding arteriole and relatively short in distance, WM capillaries are considerably longer, having to travel all the way from the GM arteriole into the adjacent WM. If this is true, then the oxygen content of blood at the distal end of these longer WM capillaries could be significantly lower than that of the relatively short GM capillaries. While our examination of the main molecular components of the BBB failed to show any major WM/GM differences in expression, this analysis was far from conclusive, and we remain mindful of the possibility that specific, yet unidentified, key molecules could be differentially expressed on blood vessels in the two types of tissue.

### Disruption of vascular integrity results in microglial activation and loss of myelin

In a recent study we showed that CMH induces vascular disruption in spinal cord blood vessels, an effect that is particularly pronounced in aged mice, and that this disruption is strongly associated with microglial activation and loss of WM myelin and oligodendroglial cells, as assessed by the marker Olig2 [16]. Our current data support these findings by showing that increased spinal cord vascular disruption leads to even greater enhancement of microglial activation and marked loss of both mature oligodendrocytes and immature oligodendrocyte precursor cells (OPCs). Notably, spinal cord vascular leak was so severe in β1 integrin-blocked mice that it resulted in very striking myelin degradation in areas of vascular leak, both in the spinal cord (Fig. 1D) and brain (Fig. 6B and D). These observations are consistent with recent work showing that fibrinogen is directly toxic to oligodendrocytes [30].

## Conclusion

In this study we demonstrate that β1 integrins play an essential role maintaining vascular integrity in the spinal cord, both under stable normoxic conditions, and even more so during hypoxia-induced vascular remodeling. Surprisingly, β1 integrin inhibition did not reduce hypoxia-induced endothelial proliferation. Notably, β1 integrin inhibition triggered much greater vascular disruption in WM, a predilection that was also observed in the brain, consistent with our observation that the rate of vascular remodeling is much greater in spinal cord WM [17]. Vascular disruption resulted in elevated microglial activation and marked loss of myelin integrity and oligodendroglial cells. Hypoxyprobe staining in spinal cord demonstrated that the most severe levels of tissue hypoxia induced by CMH occurred in the WM. These findings demonstrate a novel and unexpected difference between WM and GM blood vessels in their dependence on β1 integrin function during hypoxic exposure. They also suggest that the preferential vulnerability of WM blood vessels may be less a result of intrinsic differences in barrier properties and more a consequence of differences in vascular density and architecture.

### Electronic supplementary material

Below is the link to the electronic supplementary material.

Supplementary Material 1

## Data Availability

The datasets used and/or analysed during the current study are available from the corresponding author upon reasonable request.

## Abbreviations

BM: Basement Membrane
BBB: Blood-Brain Barrier
CMH: Chronic Mild Hypoxia
ECM: Extracellular Matrix
FOV: Field of View
GM: Grey Matter
IF: Immunofluorescence
SEM: Standard error of the mean
TJP: Tight junction protein
VCAM-1: Vascular Cell Adhesion Molecule-1
WM: White Matter
ZO-1: Zonula Occludens-1
