# Supplementary material for: β1 integrins play a critical role maintaining vascular integrity in the hypoxic spinal cord, particularly in white matter
Source: Acta Neuropathol Commun. 2024 Mar 20;12:45. doi: 10.1186/s40478-024-01749-4 (PMC10953150; doi:10.1186/s40478-024-01749-4)
Supplement: Supplementary file 1 — Supplementary Material 1 [file 40478_2024_1749_MOESM1_ESM.docx]

Supplementary Information for:

**β1 integrins play a critical role maintaining vascular integrity in the hypoxic spinal cord, particularly in white matter**

Sebok K. Halder, Arjun Sapkota, and Richard Milner

Email: rmilner@sdbri.org

**This PDF file includes:**

Supplementary Figures S1 to S4

**Figure S1**

**
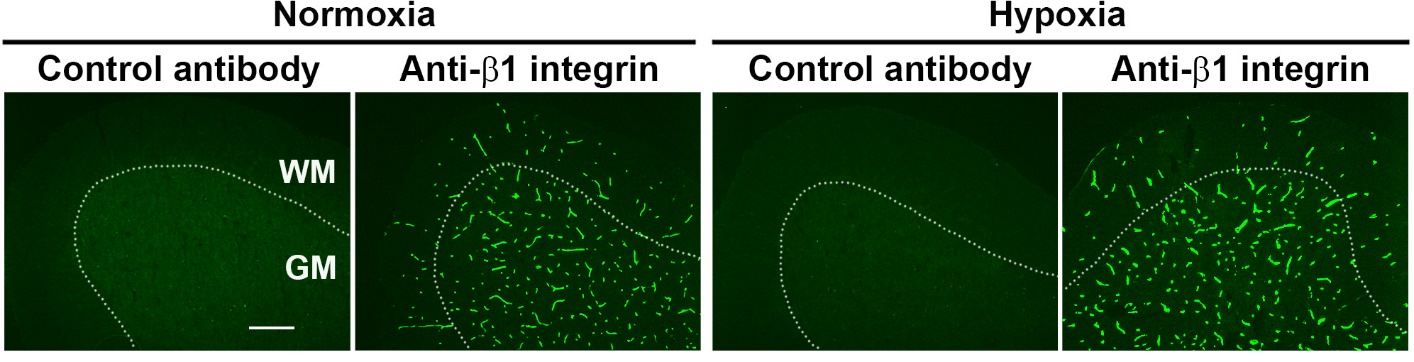
**

**Figure S1. Vascular localization of the function-blocking β1 integrin antibody in the spinal cord under normoxic and hypoxic conditions.** Frozen spinal cord sections taken from mice exposed to normoxia or hypoxia (8% O_2_) for 4 days that received daily intraperitoneal (i.p.) injections of either the anti-mouse β1 integrin function-blocking antibody HMβ1-1 or an isotype control antibody (at doses of 2.5 mg/kg) were stained with an anti-hamster secondary antibody. Scale bar = 100 μm. Note that the β1 integrin blocking antibody strongly localized to blood vessels within the white matter (WM) and grey matter (GM) of spinal cord tissue.

**Figure S2**


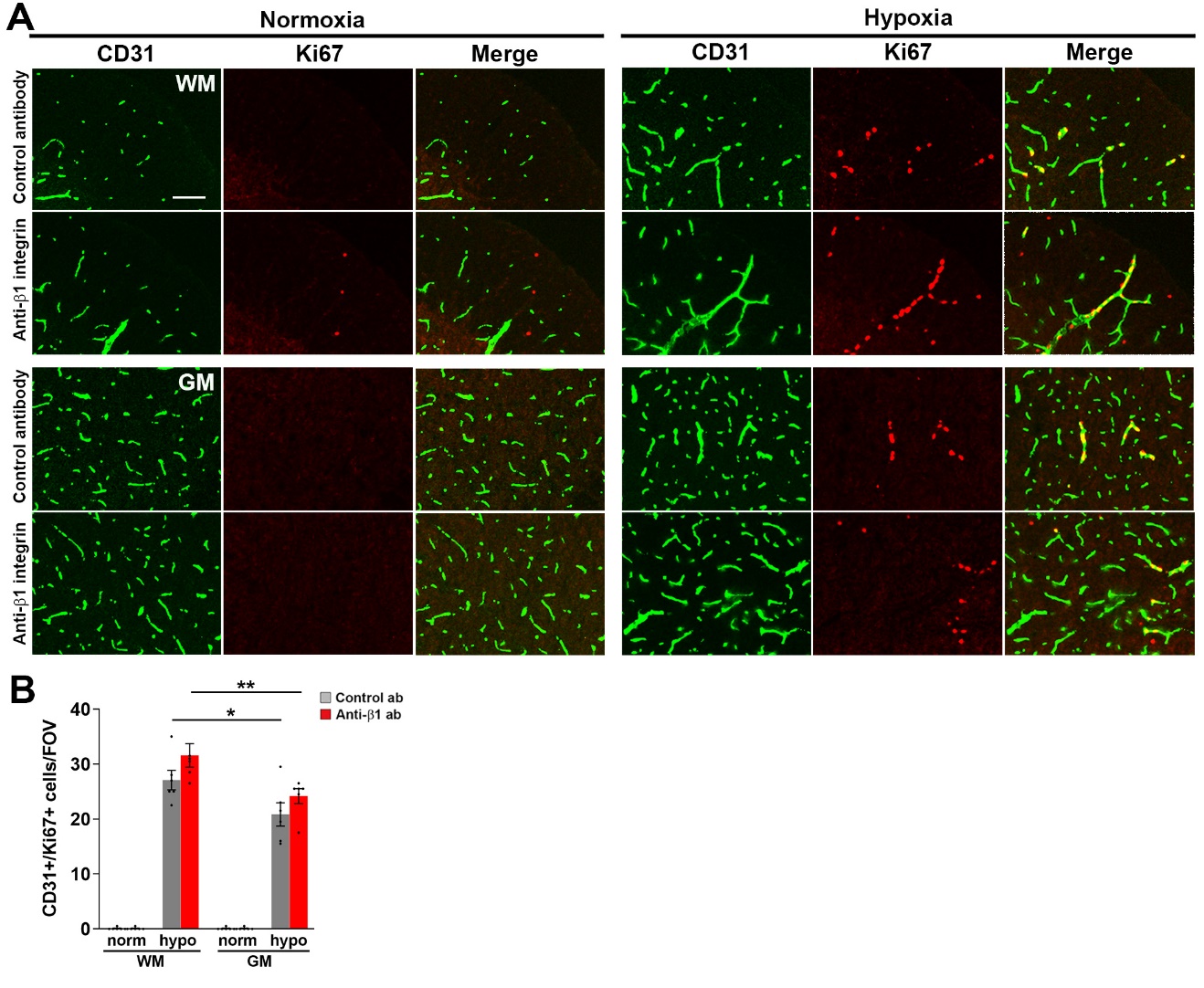


**Figure S2. β1 integrin inhibition does not prevent hypoxia-induced endothelial proliferation in the spinal cord. A.** Frozen spinal cord sections taken from mice exposed to normoxia or hypoxia (8% O_2_) that received daily intraperitoneal injections of the anti-mouse β1 integrin function-blocking antibody or isotype control antibody for 4 days were stained for CD31 (AlexaFluor-488) and the proliferation marker Ki67 (Cy-3). Scale bar = 100 μm. **B.** Quantification of the number of CD31+/ Ki67+ cells/FOV in the spinal cord after 0- or 4-days hypoxia. Results are expressed as the mean ± SEM (n = 6 mice/group). * p < 0.05, ** p < 0.01. Note that CMH strongly promoted endothelial proliferation in the spinal cord, with WM (upper two rows) showing a greater response than GM (lower two rows). Of note, β1 integrin blockade had no obvious effect on the rate of endothelial proliferation in the hypoxic spinal cord, either in WM or GM.

**Figure S3**


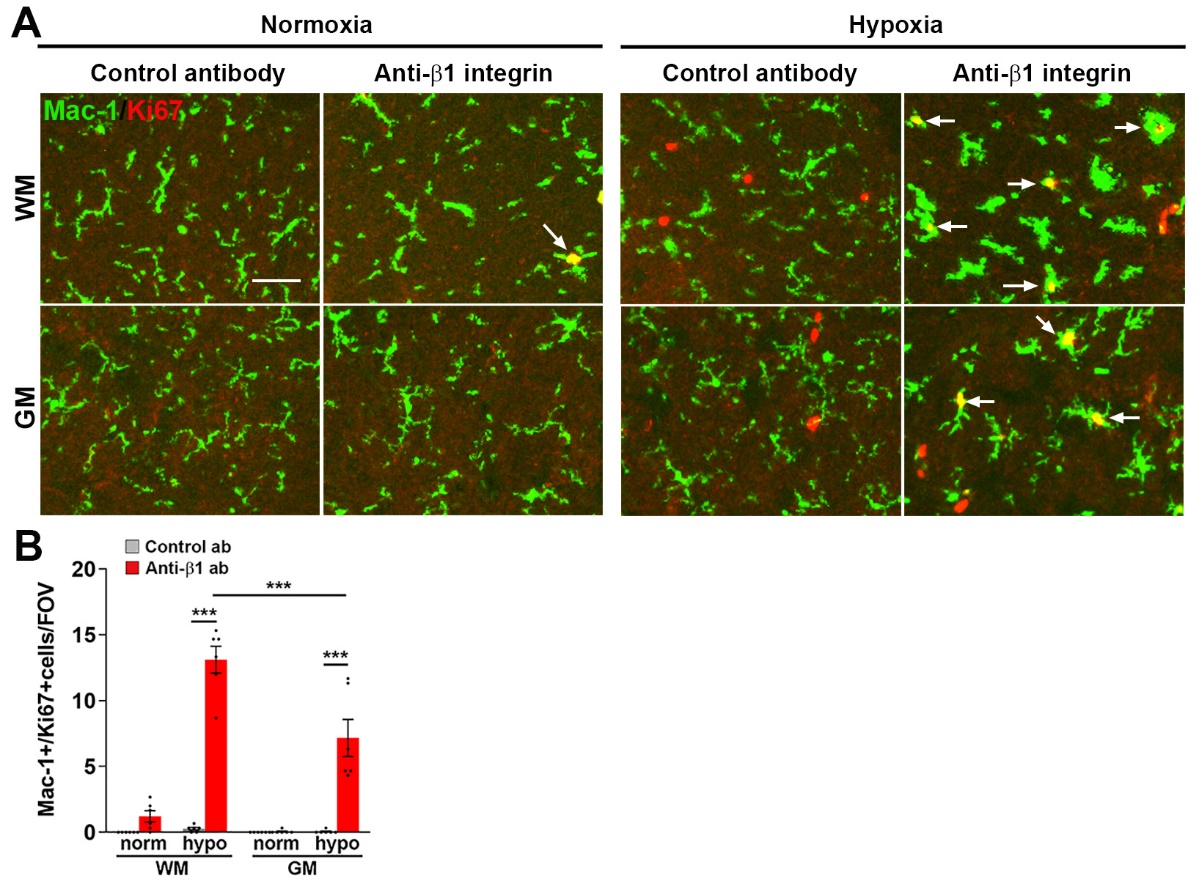


**Figure S3. β1 integrin inhibition strongly stimulated microglial proliferation in the hypoxic spinal cord.** Frozen spinal cord sections taken from mice exposed to normoxia or hypoxia (8% O_2_) that received daily intraperitoneal injections of the anti-mouse β1 integrin function-blocking antibody or isotype control antibody for 4 days were stained for Mac-1 (AlexaFluor-488) and Ki67 (Cy-3) (**A**). Scale bar = 50 μm. Arrows show Mac-1/Ki67 dual-positive proliferating microglia. **B.** Quantification of the number of Mac-1+/ Ki67+ cells/FOV in the spinal cord after 0- or 4-days hypoxia. Results are expressed as the mean ± SEM (n = 6 mice/group). *** p < 0.001. Note that β1 integrin inhibition strongly increased microglial proliferation in the hypoxic spinal cord, both in the WM and GM.

**Figure S4**


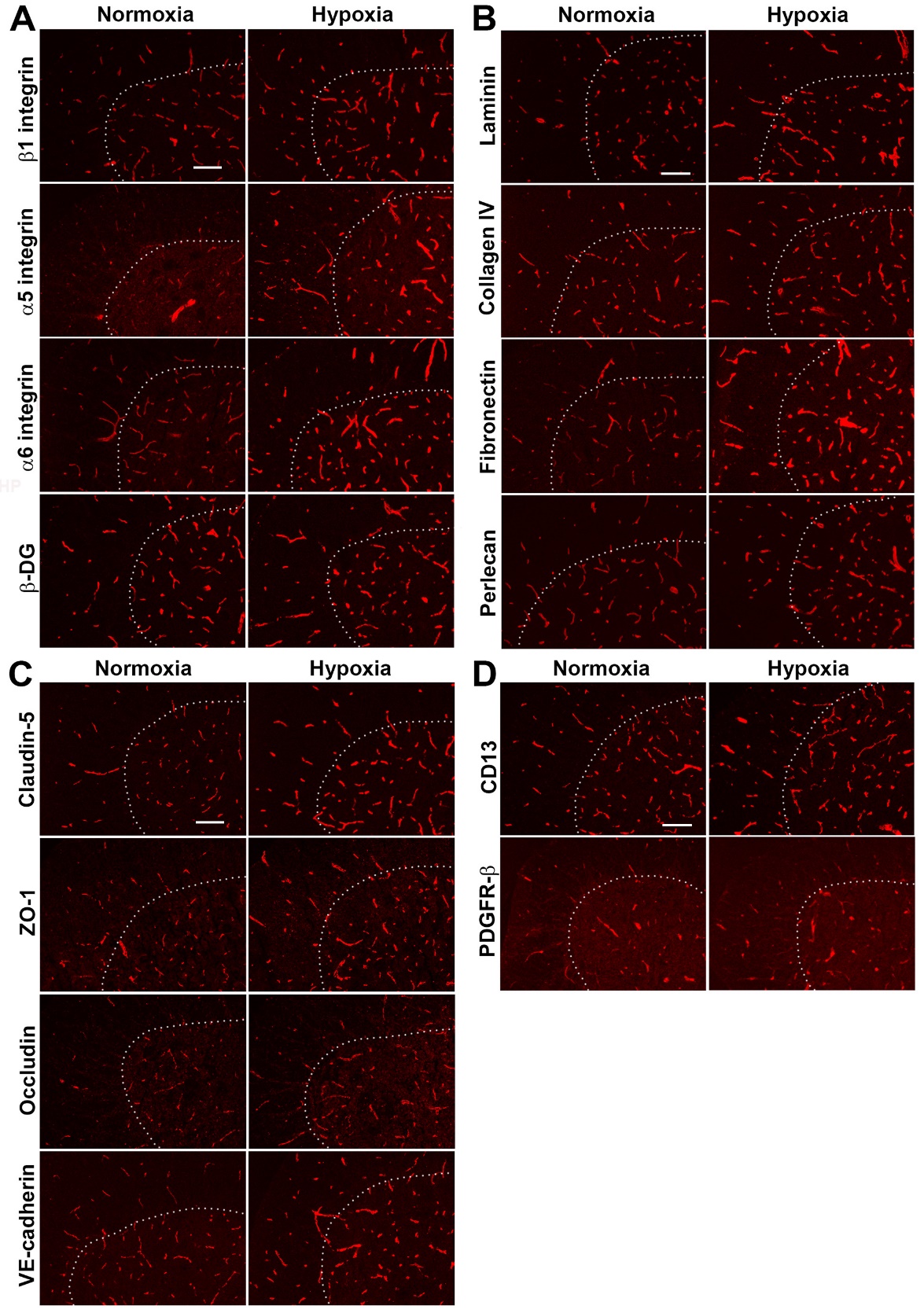


**Figure S4. Evaluation of vascular expression of adhesion and tight junction proteins and pericyte markers in spinal cord WM and GM.** Frozen spinal cord sections taken from mice exposed to normoxia or hypoxia (8% O_2_) that received daily intraperitoneal injections of the anti-mouse β1 integrin function-blocking antibody or isotype control antibody for 4 days were stained for the ECM receptors β1, α5 and α6 integrins and β-dystroglycan (β-DG) (**A**), the ECM proteins laminin, collagen IV, fibronectin, and perlecan (**B**), the tight junction proteins claudin-5, ZO-1, and occludin, and VE-cadherin (**C**), or the pericyte markers CD13 and PDGFRβ (**D**). Scale bars = 100 μm. The white dotted line demarcates the GM (inside) from the WM (outside). Note that no obvious differences in the expression level of any of these important BBB mechanisms were detected at the single vessel level.
